# Examining and Comparing the Validity and Reproducibility of Scales to Determine the Variety of Vegetables Consumed: Validation Study

**DOI:** 10.2196/55795

**Published:** 2024-04-11

**Authors:** Kaya Ominami, Osamu Kushida

**Affiliations:** 1 Department of Nutrition and Life Sciences School of Food and Nutritional Sciences University of Shizuoka Shizuoka Japan

**Keywords:** vegetable, variety, scale, validity, reproducibility, dietary records, nutrition

## Abstract

**Background:**

Previous studies have reported that vegetable variety reduces the risk for noncommunicable diseases independent of the amount consumed.

**Objective:**

This study aimed to examine and compare the validity and reproducibility of several scales to determine vegetable variety.

**Methods:**

In total, 23 nutrition students in Japan reported their vegetable intake over the past month using a self-administered questionnaire between July and August 2021. Specifically, four scales were used: (1) a single question regarding the number of vegetables consumed (scale A); (2) a scale containing 9 vegetable subgroups included in the brief-type self-administered diet history questionnaire (scale B); (3) a scale containing 19 vegetable items included in a self-administered diet history questionnaire (scale C); and (4) a scale containing 20 vegetable items from the Ranking of Vegetable Consumers in Japan, which was analyzed based on a report on the National Health and Nutrition Survey in Japan (scale D). Scale validity was assessed by correlation with the number of vegetables consumed, which was collected from dietary records for 7 consecutive days. Reproducibility was assessed by test-retest reliability.

**Results:**

Regarding the validity of the 4 scales, significant correlations were found between scales C (ρ=0.51) and D (ρ=0.44) with vegetable variety based on dietary records, but scales A (ρ=0.28) and B (ρ=0.22) were not significantly correlated. Reproducibility showed a significant correlation in scale B (ρ=0.45) and strong correlations in scales C (ρ=0.73) and D (ρ=0.75).

**Conclusions:**

The scales for vegetable items have acceptable validity and reproducibility compared to the scales that used a single question or vegetable subgroup and, therefore, may determine the variety of vegetables consumed.

## Introduction

The World Health Organization [1] recommends that adults consume at least 400 g of fruit and vegetables per day to reduce the risk of noncommunicable diseases. In Japan, the Health Japan 21 (the second term) set a consumption target for an average of 350 g of vegetables daily for adults to prevent lifestyle-related diseases [2]. However, according to the recent report of the National Health and Nutrition Survey (NHNS) in Japan, average vegetable intake is below this target at 280.5 g/d with no significant change occurring over the past 10 years [3]. This is also the case in developed countries such as the United States and Australia, and since most populations do not meet the recommended daily intake of vegetables [4,5], health policies to increase vegetable intake are being promoted worldwide.

Regarding vegetable variety, the 2020-2025 Dietary Guidelines for Americans recommend weekly intakes for vegetable subgroups [4]. The 2013 Australian Dietary Guidelines also encourage including a variety of vegetables [6]. Previous studies identified positive associations between high vegetable variety and plasma carotenoid concentrations [7] as well as overall diet quality [8]. Furthermore, vegetable variety is reported to reduce the risk of developing noncommunicable diseases such as coronary heart disease [9], lung cancer [10], and type 2 diabetes [11] independent of the amount consumed.

To measure vegetable variety, existing food frequency questionnaires (FFQs) have been used in many previous studies. However, none have examined whether the scales extracted from the FFQs reflect vegetable variety. The scales vary from study to study and are characterized by 3 main constructs: vegetable items, vegetable subgroups, or a single question [12]. Previous studies reported strong inverse associations between an increased variety of vegetable items and overall cancer risk [13] and between an increased variety of vegetable subgroups and lung cancer risk [10]. Furthermore, vegetable variety assessed by a single question has inverse associations with the risk of injurious falls and fractures [14], as well as with mortality from atherosclerotic vascular disease and common carotid artery intima-media thickness [15]. However, studies examining the vegetable variety scale itself are limited to the evaluation of a brief index of Fruit And Vegetable VAriety (FAVVA) using an FFQ in Australia [7].

Guidelines set by the United Nations Food and Agriculture Organization recommend that questionnaires assessing dietary diversity are adapted according to the culture, population, or location [16]. It is expected that developing a scale to determine vegetable variety will be important for promoting nutritional improvement in Japan. Therefore, this study aimed to examine and compare the validity and reproducibility of several scales to determine vegetable variety.

## Methods

### Study Design and Participants

This was a cross-sectional study using self-administered questionnaires. The participants were students majoring in nutrition at a university in Shizuoka, Japan, and were, therefore, familiar with dietary records. The students were recruited through a bulletin board on campus. Out of a total population of approximately 100 students, the questionnaire was distributed directly to 23 participants who agreed to be surveyed, and 23 copies were collected by mail or in person (recovery rate=100%). As an incentive, participants in this survey were given a gift certificate worth JP ¥2000 (US $14).

### Study Period

The survey period was from July to August 2021. Participants were initially asked to respond on a scale regarding the variety of vegetables consumed in the past month. Following this, participants were asked to keep dietary records for 7 consecutive days starting the day after their responses for each scale. According to a previous study that examined the number of days of dietary records required to estimate habitual vegetable variety, we estimated that 7 consecutive days of dietary records would capture approximately 70% of the maximum theoretical number of different vegetables consumed [17]. It is reasonable to assume that an understanding of dietary habits could be achieved with 7 consecutive days of dietary records; therefore, the same number of consecutive days of dietary record collection was chosen for this study. Finally, participants were asked to provide their responses on the same scale after 1 month following their response to the first scale. After each questionnaire was collected, 2 students majoring in nutrition and 1 researcher certified as a registered dietitian checked for missing questionnaire responses and incomplete dietary records (eg, missing vegetable condiments, seasonings, and cooking oil). That missing information was then obtained from participants in person or via email.

### Measurements

#### Scales to Determine Vegetable Variety

In total, 4 types of scales were used to capture responses regarding the variety of vegetables consumed in the past month.

The first scale consisted of a single question that was answered with a single or double-digit whole number, in response to the following question: “How many different vegetables do you usually eat per day?” which was adopted from an FFQ developed by the Cancer Council of Victoria [14]. This scale is defined as the variety of vegetable consumption assessed by a single question (scale A).

The second scale consisted of 9 vegetable subgroups presented in a brief-type self-administered diet history questionnaire (BDHQ) [18]. The BDHQ is a nonquantitative FFQ that has been validated for assessing vegetable intake throughout the year in urban, rural inland, and rural coastal areas in Japan (Osaka, Nagano, and Tottori) [18]. Scoring was based on the study by Chou et al [19], in which 1 point was added for consumption at least once a week, 0 for less than once a week, and the total score representing the vegetable variety of the vegetable subgroup (scale B).

The third and fourth scales consisted of 19 vegetable items presented in a self-administered diet history questionnaire (DHQ) [18], and 20 vegetable items presented in the Ranking of Vegetable Consumers in Japan report, which was analyzed based on the 2012 NHNS [20]. The DHQ is a semiquantitative FFQ that has been validated for vegetable intake throughout the year in several areas in Japan, similar to the BDHQ [18]. The Ranking of Vegetable Consumers in Japan report was determined to have good internal consistency among the 20 vegetables, according to the authors’ preliminary analyses (Cronbach α=0.78). Previous studies using vegetable items scales [10,21] add 1 point for reported consumption of at least once per 2 weeks. The same scoring was used in this study, with 0 points added for consumption that was reported as once a month or less. The total score was defined as the vegetable variety for each vegetable item (scales C and D, respectively).

The exact wording and scoring methods for each scale are shown in Multimedia Appendix 1.

#### Dietary Records for 7 Consecutive Days

Participants were provided instructions to complete their dietary records recording all foods and beverages consumed, and weighing whenever possible. Participants recorded the meal start time, a description of the meal situation (home meal, ready-made meal, eating out, or other), the name of the dish, the name of the food, and the amount consumed. The participants were asked to record the name of the product, seller, and store when consuming commercial products and the name of the restaurant when eating out. If participants recorded only the name of the dish, the food and amount consumed were estimated by referring to the top results for recipes identified through an internet search. When the name of a product or restaurant was recorded, an internet search was conducted to identify the food and amount consumed by the participant. The number of vegetables consumed was extracted from the *Standard Tables of Food Composition in Japan 2020 (Eighth Revised Edition)* [22]. In doing so, vegetables not listed were replaced with similar vegetables in the food composition tables. The Excel add-in software EiyoPlus (Kenpakusha, Co, Ltd) was used to calculate vegetable variety from the dietary records.

In this study, vegetable variety was defined as the total number of different vegetables consumed as recorded by the participant on their dietary records and the number of different vegetables consumed estimated by the methods described above. In a previous Australian study that used 24-hour recall [23], 1 unit of variety of vegetable consumption consisted of ≥50% (≥37.5 g) of a serving of a vegetable in the country. Furthermore, in a Japanese study that examined the frequency of meals including staple, main, and side dishes and nutrient intake [24], side dishes consisting mainly of vegetables were considered to have been consumed if participants consumed at least half a serving (≥35 g) in the Japanese food guide. Therefore, in this study, vegetables were counted as 1 item when the amount consumed was ≥35 g. The method for counting the number of vegetables consumed followed procedures described elsewhere [17]. Briefly, vegetables within the same category but with different food names were counted as different items, while the same vegetable prepared according to different cooking and processing methods was counted as the same item. Vegetables in the same category with different food names but prepared according to different processing methods were counted as the same item (eg, daikon, *kiriboshi-daikon* [cut and dried daikon root], and “pickles” [daikon pickled in salty rice bran paste] were counted as the same item). Furthermore, grated ginger and grated garlic were classified as spices and not vegetables.

#### Demographic Characteristics

Participants were asked about the following characteristics: gender, age, height, weight, location, number of people living together, smoking status, drinking habits, exercise habits, and whether vegetable intake was restricted for medical reasons.

### Statistical Methods

Of the 4 scales included in this study, item-total correlation analysis was performed for each of the 3 scales that are scoring systems. Dietary records for 7 consecutive days were used as a reference standard in the validity analysis. In designing a validation study for the FFQ, administering the questionnaire before the participants create dietary records results in the questionnaire responses being related to the diet before the period during which dietary records were created. On the other hand, the intensive effort involved in creating dietary records may lead to artificially improved accuracy if the questionnaire is administered after dietary record creation [25]. To avoid optimistic estimates of the correlation in this study, the first response given by participants was used to examine the validity of each scale. Reproducibility was assessed by test-retest reliability. Since each scale to determine vegetable variety was intended to be ranked, correlation coefficients were calculated. Furthermore, because the recorded variable distributions were determined not to be normally distributed by the histograms, the Spearman correlation coefficient was used to examine the validity and reproducibility. SPSS Statistics (version 27; IBM Corp) was used for analysis. The level of significance was set at *P*<.05 (2-sided test).

### Ethical Considerations

The purpose and methods of this study were explained to participants before this study. Informed consent was obtained in writing from all participants. Parental or guardian written consent was obtained from students aged 18 or 19 years. This study was approved by the Research Ethics Committee of the University of Shizuoka (3-12).

## Results

### Participants

All 23 respondents were included in the analysis. None of the participants reported restricting their vegetable intake for medical reasons.

### Demographic Characteristics

The participants were 91% (n=21) women, with a mean age of 20.0 (SD 1.4) years (Table 1).

**Table 1:** Demographic of participants (N=23).

Characteristics	Values
Women, n (%)	21 (91)
Age (years), mean (SD)	20.0 (1.4)
BMI (kg/m^2^), mean (SD)	20.6 (1.2)
From the local area, n (%)	9 (39)
Lives alone, n (%)	17 (74)
Smokes, n (%)	0 (0)
Drinks 1-3 times a month or more, n (%)	7 (30)
Exercises 1-3 times a month or more, n (%)	11 (48)

### Item Analysis

For scale B, only 1 item (“raw vegetables used in salad: lettuce, cabbage, etc”) had a correlation coefficient greater than 0.5 and was significant at the 1% level (Table 2). Correlation coefficients of 0.4 to 0.5 and significant at the 5% level were found for 2 items (“tomatoes, tomato ketchup, boiled tomato, and stewed tomato” and “vegetables used in cooking: cabbage and Chinese cabbage”).

For scale C, 4 items (“broccoli,” “lettuce,” “burdock,” and “lotus root”) had a correlation coefficient greater than 0.5 and were significant at the 1% level. Correlation coefficients of 0.4 to 0.5 and significant at the 5% level were found for 2 items (“carrots” and “pumpkins”).

For scale D, 6 items (“broccoli,” “spinach,” “Welsh onions [green],” “lettuce,” “burdock,” and “ginger”) had a correlation coefficient greater than 0.5 and were significant at the 1% level. Correlation coefficients of 0.4 to 0.5 and significant at the 5% level were found for 5 items (“carrots,” “pumpkins,” “*komatsuna*,” “Chinese chive,” and “Welsh onions [branching cultivation]”).

Since none of the items were significantly inversely correlated with the 3 scales, none of the items were removed. The validity and reproducibility of scales selected items that significantly correlated with total scores were also examined. The item selection scales were referred to as short scales B, C, and D.

**Table 2:** Item-total correlations for the 3 scored scales (N=23).

				ρ^a^		*P* value
**Scale B**						
	**Pickled vegetables**					
		Green leafy vegetables	0.23		.29	
		Other (excluding salted pickled plum)	0.23		.29	
	**Raw vegetables used in salad**					
		Lettuce, cabbage, etc (excluding tomatoes)	0.54		.008	
		Tomatoes, tomato ketchup, boiled tomato, and stewed tomato	0.55		.01	
	**Vegetables used in cooking**					
		Green leafy vegetables (including broccoli and bitter melon)	–0.05		.82	
		Cabbage and Chinese cabbage	0.45		.03	
		Carrots and pumpkins	0.23		.29	
		Daikon and turnips	0.40		.06	
		All other root vegetables (including onions, burdock, and lotus root)	0.37		.09	
**Scale C**						
	Carrots			0.47		.02
	Pumpkins			0.49		.02
	Tomatoes			0.15		.49
	Sweet peppers			0.23		.30
	Broccoli			0.67		<.001
	Green leafy vegetables			0.41		.05
	Lettuce			0.54		.008
	Cabbage			0.38		.07
	Cucumbers			0.32		.14
	Chinese cabbage			0.18		.40
	Bean sprouts			0.19		.40
	Daikon			0.32		.14
	Onions			0.36		.09
	Cauliflower			—^b^		—
	Eggplants			0.11		.63
	Burdock			0.71		<.001
	Lotus root			0.53		.009
	Pickled vegetables (excluding salted pickled plum)			0.24		.27
**Scale D**						
	Carrots			0.47		.02
	Pumpkins			0.49		.02
	Tomatoes			0.10		.69
	Sweet peppers			0.34		.11
	Broccoli			0.65		.001
	Spinach			0.78		<.001
	*Komatsuna*			0.47		.03
	Welsh onions (green)			0.62		.001
	Chinese chive			0.49		.02
	Lettuce			0.54		.008
	Welsh onions (branching cultivation)			0.47		.02
	Cabbage			0.24		.26
	Cucumbers			0.28		.20
	Chinese cabbage			0.37		.08
	Bean sprouts			0.22		.31
	Daikon			0.22		.31
	Onions			0.36		.10
	Burdock			0.74		<.001
	Ginger			0.69		<.001
	Garlic			0.08		.71

^a^Spearman correlation coefficient.

^b^Not available.

### Validity

For each scale, the correlation coefficients were as follows: scale A, ρ=0.28 (*P*=.20); scale B, ρ=0.22 (*P*=.31); scale C, ρ=0.51 (*P*=.01); and scale D, ρ=0.44 (*P*=.03; Table 3). This indicated that the 2 scales assessed by vegetable items (ie, scales C and D) were significantly correlated with vegetable variety based on the dietary records for 7 consecutive days. Furthermore, the correlation coefficient was ρ=0.08 (*P*=.73) for short scale B, ρ=0.42 (*P*=.04) for short scale C, and ρ=0.33 (*P*=.12) for short scale D, indicating that the correlation decreased with the selection of items for all 3 scales.

**Table 3:** Correlation of each scale with vegetable variety based on dietary records for 7 consecutive days (N=23).

Scale	ρ^a^	*P* value
Scale A	0.28	.20
Scale B	0.22	.31
Scale C	0.51	.01
Scale D	0.44	.03
Short scale B	0.08	.73
Short scale C	0.42	.04
Short scale D	0.33	.12

^a^Spearman correlation coefficient.

### Reproducibility

**Figure 1:**
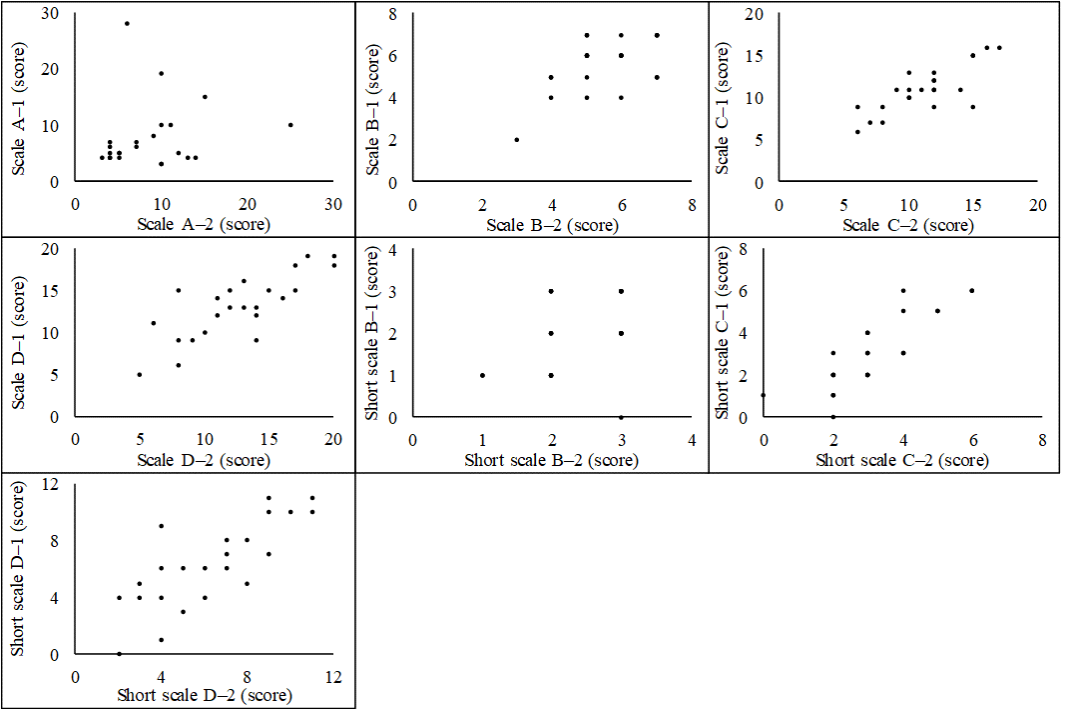
Scatter plots of the first and second responses on 4 complete scales and 3 scales with selected items (N=23).

For each scale, the correlation coefficient of the first and second responses for scale A was ρ=0.24 (*P*=.27; Figure 1). Scale B was ρ=0.45 (*P*=.03), scale C was ρ=0.73 (*P*<.001), and scale D was ρ=0.74 (*P*<.001), confirming a correlation between the 2 responses on the vegetable subgroup scale and a strong correlation for the 2 scales using vegetable items. Furthermore, short scale B was ρ=0.40 (*P*=.06), short scale C was ρ=0.85 (*P*<.001), and short scale D was ρ=0.79 (*P*<.001). Although the item selection reduced the reproducibility of the vegetable subgroup scale, the 2 scales for vegetable items were still reproducible.

## Discussion

### Principal Results and Comparison With Prior Work

In this study, the validity and reproducibility of several scales to determine vegetable variety was examined and compared. Regarding validity, scales C and D significantly correlated with vegetable variety based on dietary records for 7 consecutive days. Regarding reproducibility, a significant correlation was observed for scale B, and significant strong correlations were observed for scales C and D.

Item-total correlations were conducted to examine the internal consistency of the scales. In this analysis, none of the items in all 3 scales negatively correlated with the total score. Furthermore, although items with significant correlations were selected for each scale, correlations with vegetable variety based on dietary records for 7 consecutive days were lower than the full version of the scale. Therefore, all items removed for the short scale may be necessary for determining vegetable variety, even though their correlations are weak.

For the association with vegetable variety based on dietary records for 7 consecutive days, significant correlations (ρ>0.4) were found for scales C and D but not for scales A and B. Furthermore, for the assessment of reproducibility, significant correlations were found for scale B, and significantly strong correlations (ρ>0.7) were found for scales C and D. For validation studies of FFQs, correlation coefficients with validity lower than 0.4 are considered seriously attenuate associations [25]. Compared to the scales for a single question (scale A) and vegetable subgroup (scale B), the scales for vegetable items (scales C and D) demonstrated acceptable validity and reproducibility regarding vegetable variety and, therefore, may be useful for determining the rank of vegetable variety.

Low-intake vegetables used as condiments (eg, chopped Welsh onion as a garnish), which would have little nutritional significance, were removed from the dietary records used as a reference standard in this study with a quantitative criterion for single use, based on a previous study using the 1-day food record [24]. However, other studies that have used 24-hour recalls have not specified the minimum amount of consumption [12]. Since vegetable variety varies depending on the establishment of a minimum amount consumed using either dietary records or 24-hour recalls used as a reference standard, the necessity of establishing a minimum amount and the appropriateness of the cutoff value should be examined in the future. In this study, the scales examined did not establish a minimum amount of vegetable consumption. Nonquantitative FFQs such as the BDHQ referenced in this study, are confirmed to have reasonable validity for vegetable intake using the frequency measurements only [18]. Therefore, there appears to be little need to establish a minimum amount consumed, at least for a scale aimed at determining vegetable variety.

### Limitations

This study has some limitations, including the use of a single survey. It has been suggested that data collected on the eating patterns of individuals should include all seasons of the year as well as days in all parts of the week [26]. Indeed, a previous study reported significant seasonal differences in vegetable intake [27]. Since vegetable variety was assessed using data collected on 7 consecutive days as a reference standard, seasonality may have affected the vegetable variety measured in this study. The BDHQ and DHQ used as a reference for scales B and C have reasonable validity for vegetable intake conducted over 4 seasons [18]. Furthermore, although this study was conducted in July 2021, the Ranking of Vegetable Consumers in Japan based on the NHNS used as a reference for scale D was assessed in November 2012 [20]. Therefore, data from this study suggested that a minimal effect of seasonal differences on the ability of each scale to determine vegetable variety. A second limitation of this study is the small sample size. Since this study included only students majoring in nutrition at 1 Japanese university (mostly women, younger in age, normal or low BMI, and living alone) and interested in this survey, it is not clear to what extent the results can be generalized. The limited number of participants in this study also made it difficult to adjust for confounding factors. Future research should assess the validity of scales in different regions and populations. Although there were no missing days for the dietary records in this study, which likely occurred since the participants were students majoring in nutrition, it is suggested that the usability of the records decreases as the records progress to the seventh day [28]. A third limitation of this study is that, because biochemical indicators such as plasma carotenoid concentrations were not assessed, the effect of underreporting on the association of vegetable variety is not clear. Finally, in cases where participants ate commercial products, ate at a restaurant, or recorded only the name of the dish, the number of vegetables consumed was estimated using information from the internet. Therefore, although the results may not reflect the actual consumption of the participants in this study, generalization could be maintained because the foods that appeared most frequently on the internet were extracted.

### Conclusions

Correlation analysis of vegetable variety based on dietary records for 7 consecutive days and test-retest reliability suggests that the 2 scales for vegetable items have acceptable validity and reproducibility compared to the scales that used a single question or vegetable subgroup and, therefore, may determine the variety of vegetables consumed.

## Appendix

Multimedia Appendix 1 The exact wording and scoring methods for each scale.

## Abbreviations

BDHQ: brief-type self-administered diet history questionnaire
DHQ: diet history questionnaire
FAVVA: Fruit And Vegetable VAriety
FFQ: food frequency questionnaire
NHNS: National Health and Nutrition Survey
