# Supplementary material for: Examining and Comparing the Validity and Reproducibility of Scales to Determine the Variety of Vegetables Consumed: Validation Study
Source: JMIR Form Res. 2024 Apr 11;8:e55795. doi: 10.2196/55795 (PMC11046391; doi:10.2196/55795)
Supplement: Multimedia Appendix 1 [file formative_v8i1e55795_app1.pdf]

Table S1. The exact wording and scoring methods for each scale (responses regarding the past month).

"How many different vegetables do you usually eat per day?"[a]

"Please fill in the number(s); one number per box." ☐☐

"How often did you eat the following vegetables?"[b]	Not at all	Less than once a week	Once a week	2 to 3 times a week	4 to 6 times a week	Once a day	More than 2 times a day
**Pickled vegetables**							
Green leafy vegetables	a	b	c	d	e	f	g
Other (excluding salted pickled plum)	a	b	c	d	e	f	g
**Raw vegetables used in salad**							
Lettuce, cabbage, etc. (excluding tomatoes)	a	b	c	d	e	f	g
Tomatoes, tomato ketchup, boiled tomato, and stewed tomato	a	b	c	d	e	f	g
**Vegetables used in cooking**							
Green leafy vegetables (including broccoli and bitter melon)	a	b	c	d	e	f	g
Cabbage and Chinese cabbage	a	b	c	d	e	f	g
Carrots and pumpkins	a	b	c	d	e	f	g
Daikon and turnips	a	b	c	d	e	f	g
All other root vegetables (including onions, burdock, lotus root, etc.)	a	b	c	d	e	f	g

"How often did you eat the following vegetables?"[c]	Not at all	Once a month	2 to 3 times a month	Once a week	2 to 3 times a week	4 to 6 times a week	Once a day	More than 2 times a day
Carrots	a	b	c	d	e	f	g	h
Pumpkins	a	b	c	d	e	f	g	h
Tomatoes	a	b	c	d	e	f	g	h
Sweet peppers	a	b	c	d	e	f	g	h
Broccoli	a	b	c	d	e	f	g	h
Green leafy vegetables	a	b	c	d	e	f	g	h
Lettuce	a	b	c	d	e	f	g	h
Cabbage	a	b	c	d	e	f	g	h
Cucumbers	a	b	c	d	e	f	g	h
Chinese cabbage	a	b	c	d	e	f	g	h
Bean sprouts	a	b	c	d	e	f	g	h
Daikon	a	b	c	d	e	f	g	h
Onions	a	b	c	d	e	f	g	h
Cauliflower	a	b	c	d	e	f	g	h
Eggplants	a	b	c	d	e	f	g	h
Burdock	a	b	c	d	e	f	g	h
Lotus root	a	b	c	d	e	f	g	h
Pickled vegetables (excluding salted pickled plum)	a	b	c	d	e	f	g	h

"How often did you eat the following vegetables?"[d]	Not at all	Once a month	2 to 3 times a month	Once a week	2 to 3 times a week	4 to 6 times a week	Once a day	More than 2 times a day
Carrots	a	b	c	d	e	f	g	h
Pumpkins	a	b	c	d	e	f	g	h
Tomatoes	a	b	c	d	e	f	g	h
Sweet peppers	a	b	c	d	e	f	g	h
Broccoli	a	b	c	d	e	f	g	h
Spinach	a	b	c	d	e	f	g	h
Komatsuna	a	b	c	d	e	f	g	h
Welsh onions (green)	a	b	c	d	e	f	g	h
Chinese chive	a	b	c	d	e	f	g	h
Lettuce	a	b	c	d	e	f	g	h
Welsh onions (branching cultivation)	a	b	c	d	e	f	g	h
Cabbage	a	b	c	d	e	f	g	h
Cucumbers	a	b	c	d	e	f	g	h
Chinese cabbage	a	b	c	d	e	f	g	h
Bean sprouts	a	b	c	d	e	f	g	h
Daikon	a	b	c	d	e	f	g	h
Onions	a	b	c	d	e	f	g	h
Burdock	a	b	c	d	e	f	g	h
Ginger	a	b	c	d	e	f	g	h
Garlic	a	b	c	d	e	f	g	h

[a]Scale A: The score is single or double-digit whole number answer to the question.
[b]Scale B: The score is the total number of different vegetable subgroups eat at least once a week.
[c]Scale C: The score is the total number of different vegetable items eat at least 2 to 3 times a month.
[d]Scale D: The score is the total number of different vegetable items eat at least 2 to 3 times a month.
